# Prioritizing Disease-Related Microbes Based on the Topological Properties of a Comprehensive Network

**DOI:** 10.3389/fmicb.2021.685549

**Published:** 2021-07-08

**Authors:** Haixiu Yang, Fan Tong, Changlu Qi, Ping Wang, Jiangyu Li, Liang Cheng

**Affiliations:** ^1^College of Bioinformatics Science and Technology, Harbin Medical University, Harbin, China; ^2^Academy of Military Medical Science, Beijing, China; ^3^NHC and CAMS Key Laboratory of Molecular Probe and Targeted Theranostics, Harbin Medical University, Harbin, China

**Keywords:** microbe, disease, heterogeneous network, random walk with restart, microbe-disease associations

## Abstract

Many microbes are parasitic within the human body, engaging in various physiological processes and playing an important role in human diseases. The discovery of new microbe–disease associations aids our understanding of disease pathogenesis. Computational methods can be applied in such investigations, thereby avoiding the time-consuming and laborious nature of experimental methods. In this study, we constructed a comprehensive microbe–disease network by integrating known microbe–disease associations from three large-scale databases (Peryton, Disbiome, and gutMDisorder), and extended the random walk with restart to the network for prioritizing unknown microbe–disease associations. The area under the curve values of the leave-one-out cross-validation and the fivefold cross-validation exceeded 0.9370 and 0.9366, respectively, indicating the high performance of this method. Despite being widely studied diseases, in case studies of inflammatory bowel disease, asthma, and obesity, some prioritized disease-related microbes were validated by recent literature. This suggested that our method is effective at prioritizing novel disease-related microbes and may offer further insight into disease pathogenesis.

## Introduction

Microbial communities, including fungi, archaea, protozoa, bacteria, and viruses, are distributed across various organs of the human body, such as the skin, oral cavity, respiratory tract, and intestine (Cheng et al., 2020; Qi et al., 2021; Sommer and Backhed, 2013). It is reported that about 10^14^ microbial cells reside in the adult intestine, nearly 10 times the number of human cells. Therefore, microbes play an important role in the human body, engaging in various physiological processes, including metabolism regulation and immune defense (Das and Nair, 2019), and disorders relating to microbial communities within the human body have been linked to various human diseases (Huang et al., 2020; Yang et al., 2016). For example, Qin et al. (2010) found that inflammatory bowel disease (IBD), mainly in the forms of ulcerative colitis and Crohn’s disease, was usually caused by low microbial diversity. The diversity of the gut microbiota has also been associated with obesity, and the microbial-community composition can be intentionally manipulated to regulate the energy balance of obese individuals (Ley et al., 2005). Chen and Blaser (2007) found that colonization with *Helicobacter pylori* was inversely associated with asthma and allergy occurrence, and childhood acquisition of *H. pylori* can reduce these risks. The imbalance of microbial communities has also been associated with various types of cancer, including oral cancer (Zhang L. et al., 2019), colorectal cancer (Kim D.J. et al., 2020), and lung cancer (Zheng et al., 2020). Microbe-based disease pathogenesis is complex and can be influenced by environmental factors such as diet, smoking, and antibiotics therapy (Human Microbiome Project Consortium, 2012; Althani et al., 2016; Chen H. et al., 2017; Liu W. et al., 2020). Exploring and understanding microbe-disease associations, therefore, presents a significant challenge (Cheng et al., 2019; Cheng, 2019).

With the development of high-throughput sequencing technologies, such as 16S ribosomal RNA (16S rRNA), an increasing number of microbes have been identified, accelerating human disease research. Furthermore, projects such as the Human Microbiome Project (HMP) (Gevers et al., 2012; Nadia, and Ramana, 2020) and the Metagenomics of the Human Intestinal Tract (MetaHIT) Project^1^ were initiated to reveal the relationships between microbes and human diseases. However, traditional experimental methods for investigating microorganism-based pathogenesis are laborious and time-consuming, hindering progress in this field. In recent years, many computational methods have been successfully applied to the prediction of new associations, for example, miRNA–target association prediction (Deng et al., 2019; Yousef et al., 2007), lncRNA–target association prediction (Wang et al., 2019a; Zhang J. et al., 2019; Zhang Z. et al., 2019; Zhao et al., 2020), drug–target association prediction (Liu H. et al., 2020; Luo et al., 2017; Munir et al., 2019; Wang et al., 2020), drug–ncRNA association prediction (Yang et al., 2020), and association prediction between physical examination indicators with diabetes (Yang et al., 2021). However, these computational methods were only extended to the field of microbe–disease association prediction when the Human Microbe–Disease Association Database (HMDAD) became available (Ma et al., 2017). The HMDAD is the first resource that collects human microbe–disease associations through manual curation from 61 microbiota publications before July 2014. HMDAD documents 483 microbe–disease entries, including 39 diseases and 292 microbes, providing the foundation for subsequent computational–based microbe–disease association predictions.

Based on HMDAD, Chen X. et al. (2017) constructed a microbe–disease network and developed the KATZHMDA model for microbe–disease association prediction using the KATZ measurement and Gaussian interaction profile kernel similarity for microbes and diseases. Then, a series of computational methods were proposed to infer potential microbe–disease associations (Qu et al., 2019; Yang and Zou, 2020; Zhou et al., 2020). For example, Shen et al. (2017) extended the random walk to the microbe–disease heterogeneous network to compute the possibilities of microbe–disease associations. Huang et al. (2017) proposed NGRHMDA, which adopted neighbor-based collaborative filtering and a graph-based scoring method, to infer potential microbe–disease associations. Wang et al. developed a prediction model, NBLPIHMDA, to predict new microbe–disease associations. This model applied bidirectional label propagation on the disease similarity network and the microbe similarity network (Wang et al., 2019b). Liu Y. et al. (2020) proposed a deep matrix factorization microbe–disease association (DMFMDA) model, which combined the linear modeling ability of matrix factorization and the non-linear modeling ability of multi-layer perceptron to infer potential microbe–disease associations. To our knowledge, current computational methods for potential microbe–disease association predictions are all based on known microbe–disease associations from HMDAD. However, HMDAD documents the microbe–disease entries of only 61 publications before July 2014 and has not been updated. In recent years, research into microbe–disease associations have increased exponentially. Accordingly, some online repositories have been developed to record highly credible microbe–disease associations, such as Peryton (Skoufos et al., 2021), Disbiome (Janssens et al., 2018), and gutMDisorder (Cheng et al., 2020), which include thousands of curated microbe–disease associations.

In this study, we constructed a two-layer heterogeneous network by integrating large-scale known microbe–disease associations from the Peryton, Disbiome, and gutMDisorder databases, then extending the random walk with restart (RWR) to the network to prioritize candidate microbe–disease associations. The method fully considered the topological properties of the comprehensive network and achieved reasonable efficacy. Exploring microbe–disease relationships may not only help to reveal the mechanisms of disease pathogenesis but also provide insights to aid the prevention, diagnosis, and prognosis of various diseases.

## Materials and Methods

### Dataset Collection

The known microbe–disease associations used in this study were downloaded from the Peryton database^2^ (Skoufos et al., 2021), the Disbiome database^3^ (Janssens et al., 2018), and the gutMDisorder database^4^ (Cheng et al., 2020). Peryton is a novel resource that hosts more than 7,900 experimentally supported microbe–disease associations through manual curation of 314 publications. The database incorporates 43 diseases and 1,396 microorganisms, which are standardized *via* Medical Subject Headings (MeSH) and the NCBI Taxonomy database, respectively. Disbiome is a comprehensive database that collects microbe–disease associations from nearly 1,200 publications. Disbiome records 372 diseases and 1,622 organisms. The diseases are classified using the Medical Dictionary for Regulatory Activities (MedDRA) classification system and the microorganisms are normalized using NCBI and SILVA taxonomies. The gutMDisorder database provides a comprehensive resource for dysbiosis of the gut microbiota in disorders and interventions. gutMDisorder documents 2,263 experimentally supported microbe–disease associations between 579 gut microbes and 123 disorders or 77 intervention measures in humans. The microbes and diseases are standardized *via* the NCBI Taxonomy database and Disease Ontology (DO), respectively. The human microbe–disease associations were collected from the databases mentioned above to construct the composite heterogeneous network.

### Microbe–Disease Associations

**FIGURE 1:**
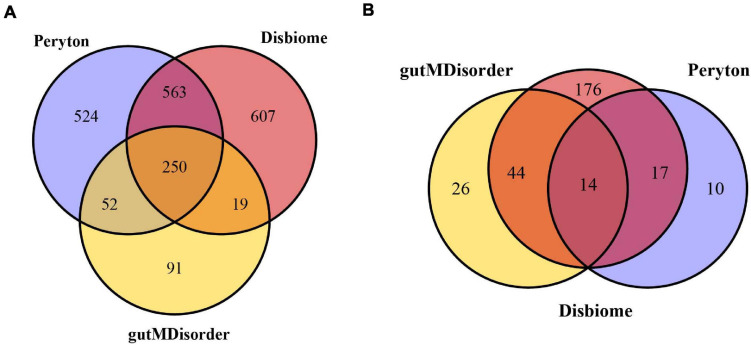
Venn diagram of overlapping microbes **(A)** and diseases **(B)** from the Peryton, Disbiome, and gutMDisorder databases.

The human microbe–disease associations were collected from the three databases mentioned above. Since the identifiers of diseases and microbes were inconsistent between different databases, we standardized the diseases and microbes *via* MeSH and the NCBI Taxonomy database, respectively. Finally, we obtained 7,810 microbe–disease associations (1,389 microbes and 41 diseases) from the Peryton database, 7,378 microbe–disease associations (1,439 microbes and 251 diseases) from the Disbiome database, and 1,249 microbe–disease associations (412 microbes and 84 diseases) from the gutMDisorder database (see Figure 1). We removed any repeated microbe–disease associations from different resources, and finally obtained 11,037 distinct microbe–disease associations involving 287 human diseases and 2,106 microbes, which were used to construct the microbe–disease network.

### Microbe Similarity

Based on the assumption that microbes with similar functions tend to share similar interactions or non-interaction patterns with diseases (Chen X. et al., 2017), we obtained the microbe similarity *via* known human microbe–disease associations using the Gaussian interaction profile kernel. The interaction profile (IP) of a microbe represented the associations between this microbe and 287 human diseases. The IP of microbe *m*_*i*_ was denoted as a vector, IP(*m*_*i*_), in which the *j*th element was set to be 1 when the disease *d*_*j*_ was confirmed to be associated with *m*_*i*_; otherwise, it was set as 0. According to the interaction profiles, the Gaussian interaction profile kernel microbe similarity was defined as follows:

(1) $$KM\left(m_{i},m_{j}\right)=exp\left(-\gamma_{m}{||IP\left(m_{i}\right)-IP\left(m_{j}\right)||}^{2}\right)$$

(2) $$\gamma_{m}=\gamma_{m}^{\prime }/\left(\frac{1}{n_{m}}\sum_{k=1}^{n_{m}}{||IP\left(m_{k}\right)||}^{2}\right)$$

In the formula mentioned above, **γ_m_** denotes the normalized kernel bandwidth, which can be calculated by a new bandwidth $\gamma_{m}^{\prime }$. In this study, we set $\gamma_{m}^{\prime }$ = 1 according to previous relevant research (Chen X. et al., 2017). **n_m_** denotes the number of microbes in this study. KM(*m*_*i*_,*m*_*j*_) denotes the Gaussian interaction profile kernel similarity between two microbes, *m*_*i*_ and *m*_*j*_. We constructed a microbe–microbe network, in which 2,106 microbes and the similarity between them were represented by nodes and edges, respectively.

### Disease Similarity

Compared with microbe similarity, disease similarity has been widely investigated. A variety of disease similarity in Cheng’s study (Cheng et al., 2018) and the Gaussian interaction profile kernel disease similarity were used in this study to obtain the disease similarity. Firstly, we calculated the Gaussian interaction profile kernel similarity between disease *d*_*i*_ and *d*_*j*_ as follows:

(3) $$KD\left(d_{i},d_{j}\right)=exp\left(-\gamma_{d}{||IP\left(d_{i}\right)-IP\left(d_{j}\right)||}^{2}\right)$$

(4) $$\gamma_{d}=\gamma_{d}^{\prime }/\left(\frac{1}{n_{d}}\sum_{k=1}^{n_{d}}{||IP\left(d_{k}\right)||}^{2}\right)$$

In the formula mentioned above, $\gamma_{d}^{\prime }$ was also set to be 1 and **n_d_** denotes the number of diseases in this study. KD(*d*_*i*_,*d*_*j*_) denotes the Gaussian interaction profile kernel similarity between two diseases, *d*_*i*_ and *d*_*j*_.

Cheng et al. (2018) provided DincRNA, a comprehensive bioinformatics resource for disease similarity calculation and non-coding RNA functional analysis. They utilized five methods, i.e., those of Wang et al. (2007), Resnik (1995), Lin (1998), PSB (Mathur and Dinakarpandian, 2012), and SemFunSim (Cheng et al., 2014) to calculate the similarity of pairwise diseases (SPWD). These methods took into consideration semantic associations, information content (IC), biological processes, and functional associations. The disease similarity score between *d*_*i*_ and *d*_*j*_ in Cheng’s study was defined as SPWD(*d*_*i*_,*d*_*j*_). Finally, the average value of Gaussian interaction profile kernel similarity as well as Cheng’s SPWD was taken as disease similarity, which is shown as follows:

(5) $$SD\left(d_{i},d_{j}\right)=\frac{KD\left(d_{i},d_{j}\right)+SPWD\left(d_{i},d_{j}\right)}{2}$$

Finally, we constructed a disease–disease network, comprising 287 human diseases, and the similarity between them was represented by edges.

### Construction of the Composite Heterogeneous Weighted Network

We constructed a composite heterogeneous weighted network by integrating the microbe–disease, microbe–microbe, and disease–disease associations mentioned above. In the composite network, there were two types of nodes, 2,106 microbes and 287 human diseases. The edges between microbes and diseases represented 11,042 distinct microbe–disease associations, and the edge weight was set to be 1 when the microbe *m*_*i*_ was confirmed to be associated with disease *d*_*j*_; otherwise, it was 0. The edges between different microbes were based on microbe similarity, and the edge weight between node *m*_*i*_ and *m*_*j*_ was denoted by KM(*m*_*i*_,*m*_*j*_). The edges between different diseases were based on disease similarity, and the edge weight between nodes *d*_*i*_ and *d*_*j*_ was denoted by SD(*d*_*i*_,*d*_*j*_).

### Prioritizing Candidate Disease-Related Microbes Based on the Composite Network

Based on the composite heterogeneous weighted network, we used the RWR to prioritize candidate disease-related microbes by fully exploiting the heterogeneous biological associations. The RWR algorithm simulates a random walker that starts from the seed nodes and then moves to their immediate neighbors or stays at the current nodes according to the probability transition matrix. The iterative transition is repeated until all vertices achieve a steady state. In this study, the formula of RWR is defined as:

(6) $$P_{t+1}=\left(1-r\right)WP_{t}+rP_{0}$$

In the abovementioned formula, *r*∈(0,1) denotes the restart probability. *P*_*t*_ denotes a vector in which the *i*th element holds the probability of being at node *i* at step *t*. *W* denotes the transition matrix, which is a column-normalized adjacency matrix of the composite network. Here, we defined the adjacency matrix W as follows:

(7) $$W=\left[\begin{array}{cc} A_{M} & B\\ B^{T} & A_{D} \end{array}\right]$$

*B* is a probability transition matrix from microbe network to disease network. Accordingly, *B*^*T*^ is the transpose of *B*. Let λ be the probability of the random walker jumping from microbe network to disease network or vice versa. We defined the transition probability from microbe network to disease network as follows:

(8) $$B_{\left(i,j\right)}=p\left(d_{j}|m_{i}\right)=\left\{ \begin{array}{c} \lambda B_{ij}/\sum_{j}B_{ij},if\sum_{j}B_{ij}\neq 0\\ 0,\text{otherwise} \end{array}\right.$$

A_*M*_ is the microbe network transition matrix. The element of *A*_*M*__(_*_*i*_*,*_*j*_*_)_ represents the probability of the random walker transition from *m*_*i*_ to *m*_*j*_, which is defined as follows:

(9) $$A_{M\left(i,j\right)}=\left\{ \begin{array}{c} \left(1-\lambda \right)M_{\left(i,j\right)}/\sum_{j}M_{\left(i,j\right)},if\sum_{j}B_{ij}\neq 0\\ M_{\left(i,j\right)}/\sum_{j}M_{\left(i,j\right)},\text{otherwise} \end{array}\right.$$

Similarly, *A*_*D*_ is the disease network transition matrix. The element of *A*_*D*_ represents the probability of the random walker transition from *d*_*i*_ to *d*_*j*_, which is defined as follows:

(10) $$A_{D\left(i,j\right)}=\left\{ \begin{array}{c} \left(1-\lambda \right)D_{\left(i,j\right)}/\sum_{j}D_{\left(i,j\right)},if\sum_{j}B_{ij}\neq 0\\ D_{\left(i,j\right)}/\sum_{j}D_{\left(i,j\right)},otherwise \end{array}\right.$$

**FIGURE 2:**
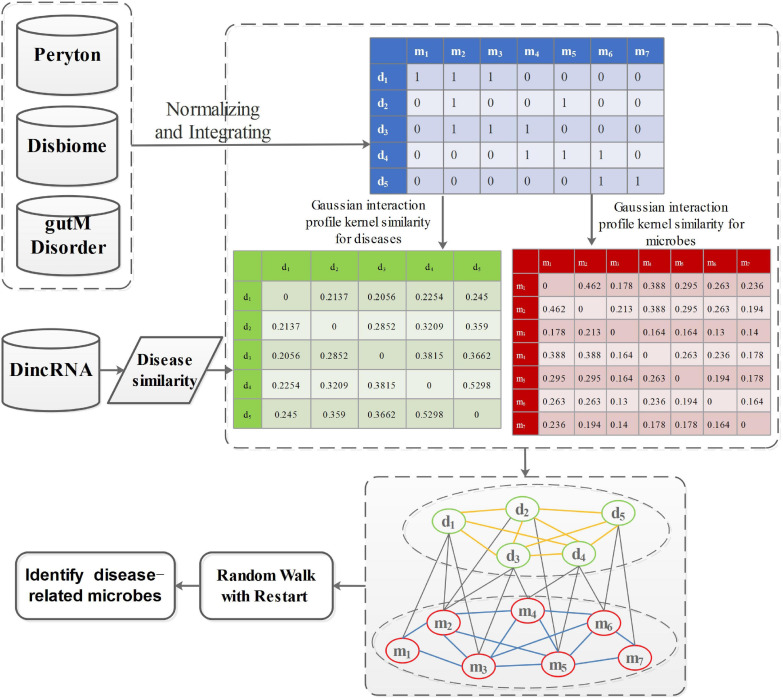
The flowchart of prioritization of candidate disease-related microbes.

*P*_0_ denotes the initial probability vector, which is a normalized unit vector. *P*_0_ = $\left[\begin{array}{c} m_{0}\\ d_{0} \end{array}\right]$ represents the initial probability vector for the heterogeneous network. *m*_0_ and *d*_0_ represent the initial probabilities of the microbe network and the disease network, respectively. After many iterations, when the difference between *P*_*t*_ and *P*_*t*__+__1_ falls below 10^–10^, it achieves a steady state. Then, microbes and diseases are ranked based on the steady probability. The flowchart of this work is shown in Figure 2.

## Results

### Performance Evaluation

To assess the performance of our method, we determined its ability to identify known disease-related microbes. The leave-one-out cross-validation (LOOCV) and fivefold cross-validation (fivefold CV) methods (Dao et al., 2020; Wang et al., 2021) were applied on known microbe–disease associations for 236 diseases, which included at least five known microbes. The receiver operating characteristic curve (ROC) plots the true-positive rate (sensitivity) versus false-positive rate (1 - specificity) at different cutoffs, and the area under the curve (AUC) was used to represent the results of cross-validation (Feng et al., 2019; Lv et al., 2020).

For LOOCV, for every disease, each known disease-related microbe was considered as one test sample, the remaining known disease-related microbes were considered as training samples, and all other unknown disease-related microbes in the composite network were considered as candidate samples. Then, we obtained a rank list of the test samples and all candidate samples according to prediction scores by performing our method. The model would achieve high prediction performance when the test samples ranked higher than the given threshold. The ROC and AUC values indicated the performance of the method. In our study, we found that all diseases achieved high predictive performance and the AUC values of LOOCV ranged from 0.9370 to 1 (see Supplementary Table 1).

For fivefold CV, for every disease, a set of known disease-related microbes was equally and randomly divided into five subparts. Each subpart was considered as the test sample in turn, and the other four subparts were considered as training samples; all of the other unknown disease-related microbes in the composite network were considered as candidate samples. Considering the potential bias caused by random sample division, we repeated this process 10 times to obtain an average AUC. Similar to LOOCV, we found that the AUC values of fivefold CV ranged from 0.9366 to 1 (Supplementary Table 2). The high predictive power indicated that the approach utilizing integrated interactions from the composite two-layer network was highly efficient in prioritizing candidate disease-related microbes.

There are two parameters in our method, one is the restart probability denoted as *r*, and the other is the probability of the random walker jumping between different networks denoted as λ. We set various values under the framework of LOOCV and fivefold CV to evaluate the impact of these parameters and found that the method achieved its best performance when *r* was set as 0.1 and λ was set as 0.5.

### Case Studies

We integrated a composite network that included 2,393 nodes (2,106 microbes and 287 human diseases) and 11,037 edges. The RWR algorithm, which makes full use of the network topology, was applied to identify candidate microbes involved in diseases among the composite network of 236 diseases. To verify the ability of our method to discover unknown associations, we implemented case studies on IBD, asthma, and obesity. The resulting list of the top 30 candidate microbes associated with these diseases is shown in Supplementary Table 3.

### Inflammatory Bowel Disease

**FIGURE 3:**
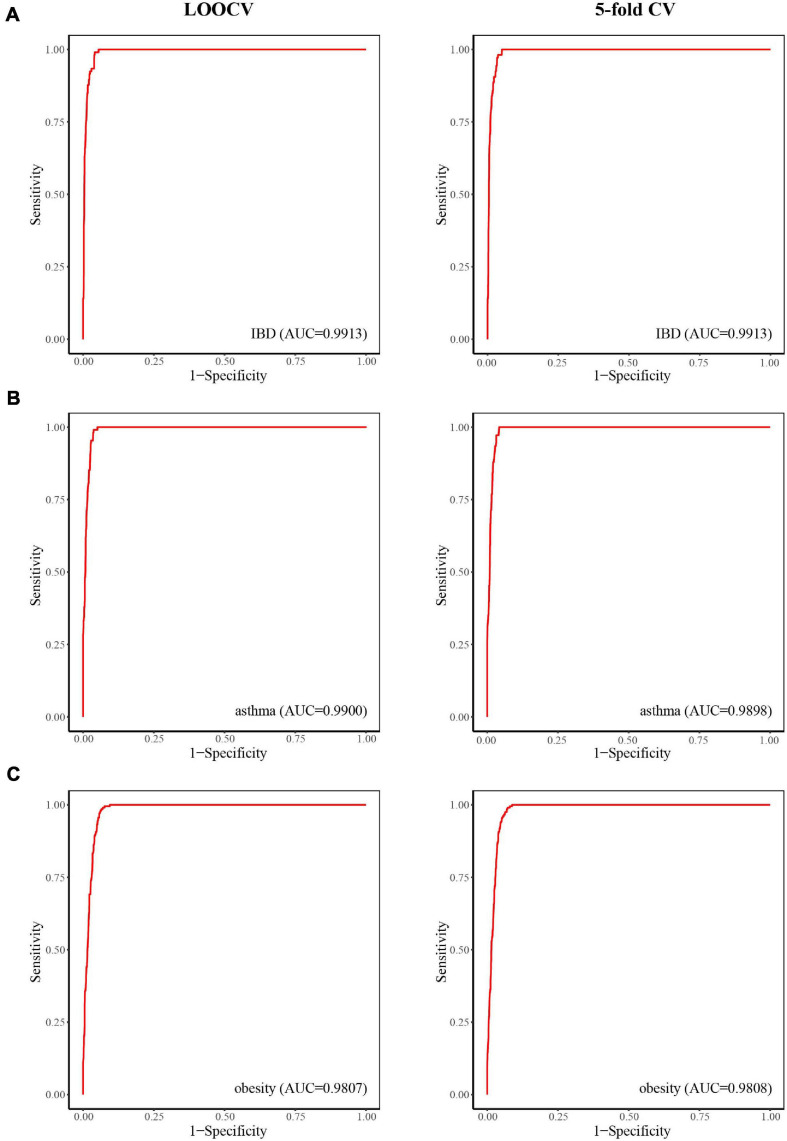
The predictive power of LOOCV (left) and fivefold CV (right) for IBD **(A)**, asthma **(B)**, and obesity **(C)**.

Inflammatory bowel disease, mainly in the form of ulcerative colitis and Crohn’s disease, is a chronic relapsing inflammatory disease of the colon and small intestine that affects an increasing number of people (Jostins et al., 2012). When considering case studies of IBD, ROC curves were obtained (Figure 3A) and the AUC values of LOOCV and fivefold CV for IBD were both 0.9913. Although there have been many studies on IBD–microbe associations (with 106 known IBD-related microbes), 16 of the top 30 prioritized IBD–microbe associations were manually confirmed by newly published literature (Table 1). For example, *Roseburia* is a top-ranked microbe in the prioritized IBD–related microbe list. Kim E.S. et al. (2020) found higher fecal calprotectin (FC) levels in pregnant patients with IBD through pregnancy, and *Roseburia* was positively correlated with maternal FC levels at T3. Sokol et al. (2018) found that IBD patients with *Clostridium difficile* infection (CDI) had more pronounced dysbiosis of *Dorea*, which was also a top-ranked microbe in the prioritized IBD-related microbe list. Toyonaga et al. (2015) found that compared with IL-10 knockout mice, the level of *Clostridium* cluster XVIII was significantly higher in OPN/IL-10 double knockout mice, when the role of osteopontin in the pathophysiology of IBD was investigated.

**TABLE 1:** Literature verification of the predicted IBD-related microbes.

**Microbe**	**Literature**
Helotiales	PMID:27811291
*Roseburia*	PMID:33307026
*Lactobacillus* sp.	PMID:30565527
*Lachnospira*	PMID:33604319
Mycobacteriaceae	PMID:32635236
*Streptococcus* sp.	PMID:19095961
Erysipelotrichaceae	PMID:33059653
*Dorea*	PMID:28786749
*Bacteroides fragilis* group	PMID:17897884
*Bacteroides stercoris*	PMID:32765449
*Akkermansia*	PMID:31892611
*Klebsiella*	PMID:32758418
*Clostridium* cluster XVIII	PMID:26274807
*Megamonas*	PMID:31776537
*Clostridium* sp.	PMID:20552029
*Fusobacterium mortiferum*	PMID:17607724

### Asthma

Asthma is a common chronic inflammatory disease caused by a variety of factors, including genetic and environment factors. Microorganisms may also play a role in the pathogenesis of asthma. Here, we considered asthma case studies, the ROC curves for which are displayed in Figure 3B, and the AUC values of LOOCV and fivefold CV for asthma were 0.9900 and 0.9898, respectively. Since asthma and its related microbes have been widely studied (with 108 known asthma-related microbes), seven of the top 30 prioritized asthma–microbe associations were manually confirmed by newly published literature (Table 2). *Blautia*, a top-ranked microbe in the prioritized IBD-related microbe list, was found to be present at high concentration in asthma patients (Fu et al., 2021). Dong et al. (2020) showed that treatment with Gu–Ben–Fang–Xiao Decoction (GBFXD) increased the abundance of Lachnospiraceae in asthmatic mice, which consequently led to elevated levels of short-chain fatty acids. Patricia et al. found that the abundance of *Epicoccum* was negatively associated with male asthma patients (Segura-Medina et al., 2019).

**TABLE 2:** Literature verification of the predicted asthma–related microbes.

**Microbe**	**Literature**
*Epicoccum*	PMID:30961954
*Galactomyces*	PMID:27711990
*Citrobacter koseri*	PMID:29062711
*Blautia*	PMID:33221308
*Clostridium* sp.	PMID:32009325
Lachnospiraceae	PMID:32431609
Unclassified Lactobacillales	PMID:27838347
	

### Obesity

Obesity is a disease associated with a body mass index of 30 kg/m^2^ or higher. It is prevalent in both adults and children worldwide and has been linked to health complications such as rheumatoid arthritis, nonallergic rhinitis, and cancer (Apovian, 2016). Here, we considered obesity case studies, the ROC curves for which are displayed in Figure 3C, and the AUC values of LOOCV and fivefold CV for obesity were 0.9807 and 0.9808, respectively. Although obesity and its related microbes have been widely studied (with 204 known obesity-related microbes), seven of the top 30 prioritized obesity–microbe associations were manually confirmed by newly published literature (Table 3). Raman et al. (2013) found that *Robinsoniella*, a top-ranked microbe in the obesity-related microbe list, was present at higher levels in nonalcoholic fatty liver disease patients and was implicated in the etiology of, and complications related to, obesity. Zeng et al. (2019) showed that *Dorea* was positively correlated with bodyweight and serum lipids, which were two significant clinical indicators of obesity.

**TABLE 3:** Literature verification of the predicted obesity-related microbes.

**Microbe**	**Literature**
Unclassified Lachnospiraceae	PMID:32784721
*Dialister succinatiphilus*	PMID:28261164
*Clostridium* cluster XVIII	PMID:31281460
rc4-4	PMID:27304513
*Dorea*	PMID:31530820
*Robinsoniella*	PMID:23454028
Enterobacteriaceae	PMID:32805279

*The case studies mentioned above indicate that our method is effective for prioritizing novel disease-related microbes, and the prioritized microbes may be used as biomarkers for disease prevention, diagnosis, and prognosis.*

## Discussion

A wide variety of microbes have been found to be parasitic within the human body. Such microbes play important roles in various physiological processes, such as metabolism regulation and immune defense. Research has also revealed that imbalances in microbial communities are closely associated with human diseases. Thus, identifying novel disease-related microbes is vital when investigating disease pathogenesis, and computational methods have been effective in achieving this. To date, the computational methods that have been applied to identify novel microbe–disease associations have all been based on the HMDAD database, which only recorded 483 microbe–disease entries from 61 publications before July 2014. In this study, we constructed a comprehensive microbe–disease network by integrating known microbe–disease associations from three novel large-scale databases (Peryton, Disbiome, and gutMDisorder), and extended the RWR to the network for prioritizing candidate disease-related microbes. The AUC values of the LOOCV and fivefold CV for 236 human diseases exceeded 0.9370 and 0.9366, respectively, indicating the high performance of our method. Furthermore, we considered case studies of IBD, asthma, and obesity. Although these three diseases have been widely studied, some prioritized disease-related microbes were validated by new publications. This finding suggested that our method is an effective method for prioritizing novel disease-related microbes, thereby aiding our understanding of disease pathogenesis.

There were some limitations in our current study. Firstly, the number of diseases considered in our study was small. This reflects the fact that large-scale microbe studies across a wide range of diseases are lacking, although the development of high-throughput sequencing technologies, such as 16S rRNA, may address this. Secondly, the microbe similarity used in this study was only based on known human microbe–disease associations using a Gaussian interaction profile kernel, which may lead to a defective heterogeneous network. This limitation may be addressed by further research into microbial functions and by integrating the functional similarities of microbes.

## Data Availability Statement

The known microbe-disease associations used in this study were downloaded from Peryton database (https://dianalab.e-ce.uth.gr/peryton/#/associations), Disbiome database (https://disbiome.ugent.be/export), and gutMDisorder database (http://bio-annotation.cn/gutMDisorder/resource.dhtml). The raw data used in this study were downloaded from the databases mentioned above, which is open source without any accession number. Other dataset presented in the study are included in the article/Supplementary Material.

## Author Contributions

LC and JL conceived and designed the study. HY and CQ collected and processed the data. HY and PW performed the experiments. HY and FT wrote the manuscript. All authors read and approved the final manuscript.

## Conflict of Interest

The authors declare that the research was conducted in the absence of any commercial or financial relationships that could be construed as a potential conflict of interest.

## Footnotes

1 http://www.metahit.eu/

2 https://dianalab.e-ce.uth.gr/peryton/

3 https://disbiome.ugent.be/home

4 http://bio-annotation.cn/gutMDisorder/home.dhtml
