# Supplementary material for: Prioritizing Disease-Related Microbes Based on the Topological Properties of a Comprehensive Network
Source: Front Microbiol. 2021 Jul 8;12:685549. doi: 10.3389/fmicb.2021.685549 (PMC8315281; doi:10.3389/fmicb.2021.685549)
Supplement: Supplementary file 1 [file Data_Sheet_1.docx]

**Table S1.** The AUC values of LOOCV for 236 diseases

| Drug | AUC |
| --- | --- |
| Acne | 0.995304 |
| Acquired Immunodeficiency Syndrome | 0.998626 |
| Acute Lymphoid Leukemia | 0.99872 |
| Acute Myeloid Leukemia | 1 |
| Aggressive Periodontitis | 0.982042 |
| Alcoholic Cirrhosis | 0.99914 |
| Alcoholic Hepatitis | 0.99641 |
| Alcoholic Pancreatitis | 0.995876 |
| Alcoholism | 0.993575 |
| Allergy | 0.999514 |
| Alopecia Areata | 0.999255 |
| Alzheimer Disease | 0.989046 |
| Amyotrophic Lateral Sclerosis | 0.996935 |
| Ankylosing Spondylitis | 0.992847 |
| Anorexia Nervosa | 0.998124 |
| Anovulation | 0.998745 |
| Anterior Uveitis | 0.99825 |
| Anti-N-Methyl-D-Aspartate Receptor Encephalitis | 0.999848 |
| Aphthous Stomatitis | 0.999682 |
| Aphthous Ulcer | 0.997671 |
| Appendicitis | 0.998668 |
| Arthritis | 0.999903 |
| Asthma | 0.990041 |
| Atherosclerosis | 0.99699 |
| Atopic Dermatitis | 0.993904 |
| Atopic Eczema | 0.999546 |
| Atrial Fibrillation | 0.998341 |
| Atrophic Gastritis | 0.998441 |
| Attention Deficit Hyperactivity Disorder | 0.997704 |
| Autism Spectrum Disorder | 0.987501 |
| Autistic Disorder | 0.99054 |
| Autoimmune Hepatitis | 0.999354 |
| Autoimmune Thrombocytopenia | 1 |
| Azoospermia | 0.999794 |
| Bacterial Vaginosis | 0.992859 |
| Barrett Esophagus | 0.998102 |
| Behcet Disease | 0.996329 |
| Biliary Atresia | 0.998359 |
| Biliary Tract Neoplasms | 0.999924 |
| Bipolar Disorder | 0.999244 |
| Bladder Neoplasms | 0.995504 |
| Blepharitis | 0.999981 |
| Breast Neoplasms | 0.989004 |
| Bronchiectasis | 0.997194 |
| Budd-Chiari Syndrome | 0.999905 |
| Bullous Pemphigoid | 0.999524 |
| Celiac Disease | 0.987844 |
| Cervical Neoplasms | 0.994719 |
| Chagas Disease | 0.999009 |
| Chlamydia | 0.991012 |
| Cholecystitis | 0.999517 |
| Cholestasis | 0.997858 |
| Chronic Fatigue Syndrome | 0.998828 |
| Chronic Kidney Diseases | 0.988803 |
| Chronic Obstructive Pulmonary Disease | 0.995986 |
| Chronic Pancreatitis | 0.998995 |
| Chronic Urticaria | 0.999894 |
| Cirrhosis | 0.986656 |
| Clonorchis sinensis Infection | 0.998689 |
| Clostridioides difficile | 0.99939 |
| Clostridium difficile Infection | 0.993562 |
| Collagenous Colitis | 0.99918 |
| Colon Neoplasms | 0.99767 |
| Colorectal Neoplasms | 0.937066 |
| Common Variable Immunodeficiency | 0.999764 |
| Constipation | 0.994367 |
| Coronary Artery Disease | 0.990124 |
| Coronary Heart Disease | 0.997112 |
| COVID-19 | 0.998026 |
| Critical Illness | 1 |
| Crohn Disease | 0.941544 |
| Cystic Fibrosis | 0.984505 |
| Dandruff | 0.997046 |
| Dental Caries | 0.986824 |
| Denture Stomatitis | 0.999638 |
| Diarrhea | 0.996098 |
| Diverticular Diseases | 0.99983 |
| Down Syndrome | 0.999955 |
| Drug Resistant Epilepsy | 0.999011 |
| Dry Eye Syndromes | 0.999048 |
| Dyspepsia | 0.999218 |
| Eczema | 0.99953 |
| End-Stage Renal Disease | 0.997608 |
| Endometrial Neoplasms | 0.998172 |
| Endometriosis | 0.996268 |
| Eosinophilic Esophagitis | 0.999222 |
| Epilepsy | 0.999669 |
| Esophageal Neoplasms | 0.996112 |
| Fatigue | 1 |
| Fatty Liver | 0.996911 |
| Fibromyalgia | 0.995649 |
| Food Allergy | 0.999771 |
| Frail Elderly | 0.999604 |
| Frailty | 0.999543 |
| Gallbladder Neoplasms | 0.999641 |
| Gastric Neoplasms | 0.997274 |
| Gastroenteritis | 0.998308 |
| Gestational Diabetes | 0.993563 |
| Glycogen Storage Disease | 0.998699 |
| Granulomatosis with Polyangiitis | 1 |
| Graves Disease | 0.99961 |
| Graves Ophthalmopathy | 0.999373 |
| Halitosis | 0.995962 |
| Hashimoto Thyroiditis | 0.998435 |
| Head and Neck Neoplasms | 0.996676 |
| Heart Failure | 0.998156 |
| Henoch-Schoenlein Purpura | 0.999924 |
| Hepatic Encephalopathy | 0.995318 |
| Hepatitis B | 0.995799 |
| Hepatitis C | 0.997956 |
| Hidradenitis Suppurativa | 0.998345 |
| Hirschsprung Disease | 0.999791 |
| HIV Infections | 0.985672 |
| Huntington Disease | 0.997865 |
| Hyperglycemia | 0.999922 |
| Hypertension | 0.993302 |
| Hyperthyroidism | 0.999524 |
| Hypopharyngeal Neoplasms | 0.99979 |
| Idiopathic Pulmonary Fibrosis | 0.999822 |
| Infantile Eczema | 0.994904 |
| Inflammatory Bowel Disease | 0.991364 |
| Interstitial Cystitis | 0.998334 |
| Intrahepatic Cholangiocarcinoma | 0.999656 |
| Iron-Deficiency Anemia | 0.99895 |
| Irritable Bowel Syndrome | 0.9767 |
| Ischemic Stroke | 0.999466 |
| Juvenile Idiopathic Arthritis | 0.998676 |
| Kaposi Sarcoma | 0.999436 |
| Kawasaki Disease | 0.999092 |
| Keratitis | 0.99857 |
| Kidney Stones | 0.997938 |
| Laryngeal Neoplasms | 0.996598 |
| Liver Cirrhosis | 0.998331 |
| Liver Neoplasms | 0.983958 |
| Lung Diseases | 1 |
| Lung Neoplasms | 0.986629 |
| Macular Degeneration | 0.999615 |
| Major Depressive Disorder | 0.996447 |
| Meningococcal Disease | 0.999173 |
| Metabolic Syndrome | 0.997975 |
| Microscopic Colitis | 0.997761 |
| Migraine | 0.997023 |
| Mouth Neoplasms | 0.98877 |
| Mucosa-Associated Lymphoid Tissue Lymphoma | 0.998303 |
| Multiple Sclerosis | 0.993401 |
| Multiple System Atrophy | 0.99828 |
| Myasthenia Gravis | 0.998502 |
| Necrotizing Enterocolitis | 0.997469 |
| Neisseria gonorrhoeae Infection | 0.999882 |
| Nephrolithiasis | 0.997511 |
| Neuromyelitis Optica | 0.998696 |
| Non-alcoholic Fatty Liver Disease | 0.988262 |
| Obesity | 0.980789 |
| Opisthorchis felineus Infection | 0.999448 |
| Oral Leukoplakia | 0.999621 |
| Oral Lichen Planus | 0.99679 |
| Oral Neoplasms | 0.995503 |
| Oropharyngeal Neoplasms | 0.999502 |
| Osteoarthritis | 0.999553 |
| Osteoporosis | 0.999729 |
| Otitis Media | 0.997049 |
| Ovarian Neoplasms | 0.999337 |
| Overactive Bladder | 0.997711 |
| Pancreatic Head Cancer | 0.997747 |
| Pancreatic Neoplasms | 0.988997 |
| Pancreatitis | 0.997781 |
| Parkinson Disease | 0.972393 |
| Pediculosis | 1 |
| Pelvic Inflammatory Disease | 0.998862 |
| Pemphigus Vulgaris | 0.999523 |
| Periodontal Abscess | 0.998573 |
| Periodontal Diseases | 0.988328 |
| Periodontitis | 0.989527 |
| Pharyngeal Neoplasms | 0.997242 |
| Phenylketonurias | 0.998785 |
| Pneumococcal Diseases | 0.998962 |
| Pneumonia | 0.99855 |
| Polycystic Ovary Syndrome | 0.996987 |
| Polyposis Coli | 0.99789 |
| Postmenopause | 0.998153 |
| Pouchitis | 0.997808 |
| Pre-Eclampsia | 0.996918 |
| Prediabetes | 0.999733 |
| Pregnancy | 0.99277 |
| Premature Birth | 0.993005 |
| Pressure Ulcer | 0.999673 |
| Primary Biliary Cholangitis | 0.996865 |
| Primary Biliary Cirrhosis | 0.998237 |
| Primary Open Angle Glaucoma | 0.999312 |
| Primary Sclerosing Cholangitis | 0.993474 |
| Prostatic Neoplasms | 0.998955 |
| Prostatitis | 0.998781 |
| Psoriasis | 0.990921 |
| Psoriatic Arthritis | 1 |
| Pulmonary Arterial Hypertension | 0.998951 |
| Pulmonary Tuberculosis | 0.994317 |
| Reactive Arthritis | 0.999206 |
| Rectal Neoplasms | 0.996286 |
| Recurrent Miscarriage | 0.999943 |
| Rett Syndrome | 0.996218 |
| Rheumatoid Arthritis | 0.993403 |
| Rosacea | 1 |
| Schizophrenia | 0.990581 |
| Seborrheic Dermatitis | 0.996567 |
| Severe Acute Malnutrition | 1 |
| Severe Fever with Thrombocytopenia Syndrome | 0.99859 |
| Short Bowel Syndrome | 0.993791 |
| Sickle Cell Disease | 0.99996 |
| Silicosis | 0.999228 |
| Sjogren's Syndrome | 0.996484 |
| Smoking | 0.986155 |
| Spinal Cord Injuries | 0.999061 |
| Squamous Intraepithelial Lesions | 0.999471 |
| Stevens-Johnson Syndrome | 0.994511 |
| Stomach Neoplasms | 0.983423 |
| Systemic Lupus Erythematosus | 0.995969 |
| Systemic Sclerosis | 0.997791 |
| Thyroid Neoplasms | 0.995024 |
| Thyroiditis | 0.99865 |
| Tongue Neoplasms | 0.996406 |
| Tonsillitis | 0.9979 |
| Transient Ischemic Attack | 0.998391 |
| Tuberculosis | 0.999448 |
| Type 1 Diabetes Mellitus | 0.993594 |
| Type 2 Diabetes Mellitus | 0.981288 |
| Ulcerative Colitis | 0.972148 |
| Urinary Bladder Neoplasms | 0.996315 |
| Urinary Urge Incontinence | 0.997867 |
| Urogenital Schistosomiasis | 0.996969 |
| Uterine Cervical Neoplasms | 0.995092 |
| Uterine Cervicitis | 0.997022 |
| Vascular Stiffness | 0.999314 |
| Vitiligo | 0.999036 |
| Vulvar Vestibulitis Syndrome | 0.997909 |
| Wilson Disease | 0.999779 |
| Wiskott-Aldrich Syndrome | 0.999933 |

**Table S2.** The AUC values of 5-fold CV for 236 diseases

| Drug | AUC |
| --- | --- |
| Acne | 0.994915 |
| Acquired Immunodeficiency Syndrome | 0.998715 |
| Acute Lymphoid Leukemia | 0.99884 |
| Acute Myeloid Leukemia | 1 |
| Aggressive Periodontitis | 0.982183 |
| Alcoholic Cirrhosis | 0.999162 |
| Alcoholic Hepatitis | 0.996577 |
| Alcoholic Pancreatitis | 0.996276 |
| Alcoholism | 0.993576 |
| Allergy | 0.999638 |
| Alopecia Areata | 0.999375 |
| Alzheimer Disease | 0.988743 |
| Amyotrophic Lateral Sclerosis | 0.997133 |
| Ankylosing Spondylitis | 0.993118 |
| Anorexia Nervosa | 0.998223 |
| Anovulation | 0.998696 |
| Anterior Uveitis | 0.997963 |
| Anti-N-Methyl-D-Aspartate Receptor Encephalitis | 0.999848 |
| Aphthous Stomatitis | 0.999692 |
| Aphthous Ulcer | 0.997733 |
| Appendicitis | 0.998595 |
| Arthritis | 0.99992 |
| Asthma | 0.989799 |
| Atherosclerosis | 0.996733 |
| Atopic Dermatitis | 0.993856 |
| Atopic Eczema | 0.999523 |
| Atrial Fibrillation | 0.998535 |
| Atrophic Gastritis | 0.998465 |
| Attention Deficit Hyperactivity Disorder | 0.997848 |
| Autism Spectrum Disorder | 0.987532 |
| Autistic Disorder | 0.990499 |
| Autoimmune Hepatitis | 0.999374 |
| Autoimmune Thrombocytopenia | 1 |
| Azoospermia | 0.999366 |
| Bacterial Vaginosis | 0.992693 |
| Barrett Esophagus | 0.998141 |
| Behcet Disease | 0.996091 |
| Biliary Atresia | 0.998556 |
| Biliary Tract Neoplasms | 0.999924 |
| Bipolar Disorder | 0.999376 |
| Bladder Neoplasms | 0.995788 |
| Blepharitis | 0.999981 |
| Breast Neoplasms | 0.988405 |
| Bronchiectasis | 0.997345 |
| Budd-Chiari Syndrome | 0.999905 |
| Bullous Pemphigoid | 0.999555 |
| Celiac Disease | 0.987436 |
| Cervical Neoplasms | 0.994795 |
| Chagas Disease | 0.998702 |
| Chlamydia | 0.991096 |
| Cholecystitis | 0.999576 |
| Cholestasis | 0.997908 |
| Chronic Fatigue Syndrome | 0.998876 |
| Chronic Kidney Diseases | 0.988939 |
| Chronic Obstructive Pulmonary Disease | 0.995419 |
| Chronic Pancreatitis | 0.999107 |
| Chronic Urticaria | 0.999892 |
| Cirrhosis | 0.986544 |
| Clonorchis sinensis Infection | 0.998727 |
| Clostridioides difficile | 0.999102 |
| Clostridium difficile Infection | 0.993897 |
| Collagenous Colitis | 0.999293 |
| Colon Neoplasms | 0.997963 |
| Colorectal Neoplasms | 0.93664 |
| Common Variable Immunodeficiency | 0.999764 |
| Constipation | 0.994236 |
| Coronary Artery Disease | 0.990479 |
| Coronary Heart Disease | 0.997148 |
| COVID-19 | 0.997774 |
| Critical Illness | 1 |
| Crohn Disease | 0.941195 |
| Cystic Fibrosis | 0.984401 |
| Dandruff | 0.996792 |
| Dental Caries | 0.986715 |
| Denture Stomatitis | 0.999638 |
| Diarrhea | 0.996271 |
| Diverticular Diseases | 0.999832 |
| Down Syndrome | 0.999958 |
| Drug Resistant Epilepsy | 0.998964 |
| Dry Eye Syndromes | 0.999131 |
| Dyspepsia | 0.999336 |
| Eczema | 0.999036 |
| End-Stage Renal Disease | 0.997877 |
| Endometrial Neoplasms | 0.998253 |
| Endometriosis | 0.996012 |
| Eosinophilic Esophagitis | 0.999381 |
| Epilepsy | 0.999632 |
| Esophageal Neoplasms | 0.996484 |
| Fatigue | 0.999995 |
| Fatty Liver | 0.99692 |
| Fibromyalgia | 0.995822 |
| Food Allergy | 0.999771 |
| Frail Elderly | 0.999686 |
| Frailty | 0.999543 |
| Gallbladder Neoplasms | 0.99957 |
| Gastric Neoplasms | 0.997387 |
| Gastroenteritis | 0.99846 |
| Gestational Diabetes | 0.993645 |
| Glycogen Storage Disease | 0.998959 |
| Granulomatosis with Polyangiitis | 1 |
| Graves Disease | 0.999654 |
| Graves Ophthalmopathy | 0.99947 |
| Halitosis | 0.995826 |
| Hashimoto Thyroiditis | 0.998505 |
| Head and Neck Neoplasms | 0.996847 |
| Heart Failure | 0.998127 |
| Henoch-Schoenlein Purpura | 0.999924 |
| Hepatic Encephalopathy | 0.995378 |
| Hepatitis B | 0.99625 |
| Hepatitis C | 0.99806 |
| Hidradenitis Suppurativa | 0.998497 |
| Hirschsprung Disease | 0.999739 |
| HIV Infections | 0.985652 |
| Huntington Disease | 0.998151 |
| Hyperglycemia | 0.999958 |
| Hypertension | 0.993352 |
| Hyperthyroidism | 0.999622 |
| Hypopharyngeal Neoplasms | 0.999788 |
| Idiopathic Pulmonary Fibrosis | 0.999883 |
| Infantile Eczema | 0.995246 |
| Inflammatory Bowel Disease | 0.991308 |
| Interstitial Cystitis | 0.99797 |
| Intrahepatic Cholangiocarcinoma | 0.999686 |
| Iron-Deficiency Anemia | 0.99912 |
| Irritable Bowel Syndrome | 0.976532 |
| Ischemic Stroke | 0.999417 |
| Juvenile Idiopathic Arthritis | 0.998725 |
| Kaposi Sarcoma | 0.999491 |
| Kawasaki Disease | 0.999141 |
| Keratitis | 0.997616 |
| Kidney Stones | 0.997945 |
| Laryngeal Neoplasms | 0.997003 |
| Liver Cirrhosis | 0.998508 |
| Liver Neoplasms | 0.983852 |
| Lung Diseases | 1 |
| Lung Neoplasms | 0.986309 |
| Macular Degeneration | 0.999506 |
| Major Depressive Disorder | 0.996818 |
| Meningococcal Disease | 0.999202 |
| Metabolic Syndrome | 0.997712 |
| Microscopic Colitis | 0.997872 |
| Migraine | 0.997149 |
| Mouth Neoplasms | 0.988569 |
| Mucosa-Associated Lymphoid Tissue Lymphoma | 0.997616 |
| Multiple Sclerosis | 0.993317 |
| Multiple System Atrophy | 0.998114 |
| Myasthenia Gravis | 0.998674 |
| Necrotizing Enterocolitis | 0.997736 |
| Neisseria gonorrhoeae Infection | 0.999881 |
| Nephrolithiasis | 0.997758 |
| Neuromyelitis Optica | 0.998556 |
| Non-alcoholic Fatty Liver Disease | 0.988284 |
| Obesity | 0.98083 |
| Opisthorchis felineus Infection | 0.99937 |
| Oral Leukoplakia | 0.999653 |
| Oral Lichen Planus | 0.996421 |
| Oral Neoplasms | 0.995363 |
| Oropharyngeal Neoplasms | 0.999425 |
| Osteoarthritis | 0.999632 |
| Osteoporosis | 0.999756 |
| Otitis Media | 0.996947 |
| Ovarian Neoplasms | 0.998793 |
| Overactive Bladder | 0.997705 |
| Pancreatic Head Cancer | 0.997569 |
| Pancreatic Neoplasms | 0.989104 |
| Pancreatitis | 0.998038 |
| Parkinson Disease | 0.972098 |
| Pediculosis | 1 |
| Pelvic Inflammatory Disease | 0.998895 |
| Pemphigus Vulgaris | 0.999534 |
| Periodontal Abscess | 0.998757 |
| Periodontal Diseases | 0.988422 |
| Periodontitis | 0.989438 |
| Pharyngeal Neoplasms | 0.997001 |
| Phenylketonurias | 0.998972 |
| Pneumococcal Diseases | 0.999162 |
| Pneumonia | 0.998628 |
| Polycystic Ovary Syndrome | 0.997181 |
| Polyposis Coli | 0.998049 |
| Postmenopause | 0.99805 |
| Pouchitis | 0.99755 |
| Pre-Eclampsia | 0.997038 |
| Prediabetes | 0.999733 |
| Pregnancy | 0.992842 |
| Premature Birth | 0.993222 |
| Pressure Ulcer | 0.999583 |
| Primary Biliary Cholangitis | 0.997177 |
| Primary Biliary Cirrhosis | 0.998283 |
| Primary Open Angle Glaucoma | 0.999395 |
| Primary Sclerosing Cholangitis | 0.993713 |
| Prostatic Neoplasms | 0.997956 |
| Prostatitis | 0.998784 |
| Psoriasis | 0.990819 |
| Psoriatic Arthritis | 0.999992 |
| Pulmonary Arterial Hypertension | 0.998936 |
| Pulmonary Tuberculosis | 0.994161 |
| Reactive Arthritis | 0.999363 |
| Rectal Neoplasms | 0.996214 |
| Recurrent Miscarriage | 0.999943 |
| Rett Syndrome | 0.996546 |
| Rheumatoid Arthritis | 0.993603 |
| Rosacea | 1 |
| Schizophrenia | 0.990507 |
| Seborrheic Dermatitis | 0.996726 |
| Severe Acute Malnutrition | 1 |
| Severe Fever with Thrombocytopenia Syndrome | 0.998863 |
| Short Bowel Syndrome | 0.994294 |
| Sickle Cell Disease | 0.999962 |
| Silicosis | 0.999217 |
| Sjogren's Syndrome | 0.996113 |
| Smoking | 0.986737 |
| Spinal Cord Injuries | 0.999168 |
| Squamous Intraepithelial Lesions | 0.999525 |
| Stevens-Johnson Syndrome | 0.994591 |
| Stomach Neoplasms | 0.983302 |
| Systemic Lupus Erythematosus | 0.995748 |
| Systemic Sclerosis | 0.997613 |
| Thyroid Neoplasms | 0.995348 |
| Thyroiditis | 0.998744 |
| Tongue Neoplasms | 0.996445 |
| Tonsillitis | 0.997971 |
| Transient Ischemic Attack | 0.998643 |
| Tuberculosis | 0.999493 |
| Type 1 Diabetes Mellitus | 0.993633 |
| Type 2 Diabetes Mellitus | 0.98116 |
| Ulcerative Colitis | 0.97184 |
| Urinary Bladder Neoplasms | 0.996191 |
| Urinary Urge Incontinence | 0.997954 |
| Urogenital Schistosomiasis | 0.997311 |
| Uterine Cervical Neoplasms | 0.994903 |
| Uterine Cervicitis | 0.997075 |
| Vascular Stiffness | 0.999314 |
| Vitiligo | 0.99921 |
| Vulvar Vestibulitis Syndrome | 0.998137 |
| Wilson Disease | 0.999776 |
| Wiskott-Aldrich Syndrome | 0.99992 |

**Table S3.** Prediction results of the top 30 disease-related microbes

|  | IBD | Asthma | Obesity |
| --- | --- | --- | --- |
| 1 | Helotiales | Dialister succinatiphilus | Brachybacterium |
| 2 | Stramenopiles | Clostridium cluster XlVa | unclassified Lachnospiraceae |
| 3 | Byssochlamys | Brachybacterium | Neisseria bacilliformis |
| 4 | Clostridium cellulosi | Byssochlamys | Dialister succinatiphilus |
| 5 | unclassified Lachnospiraceae | Stramenopiles | Lactobacillus rogosae |
| 6 | Bacteroides nordii | Helotiales | Desulfotomaculum |
| 7 | Brachybacterium | Clostridium cluster XIVb | Byssochlamys |
| 8 | Roseburia | Citricoccus | Stramenopiles |
| 9 | Paraprevotella xylaniphila | Oceanobacillus | Helotiales |
| 10 | Aquabacterium parvum | Epicoccum | Bacteroides nordii |
| 11 | Lactobacillus sp. | Galactomyces | Clostridium cluster XVIII |
| 12 | Galactomyces | Citrobacter koseri | Actinomyces lingnae |
| 13 | Citrobacter koseri | Kineothrix | Aquabacterium parvum |
| 14 | Epicoccum | Aquabacterium parvum | Caldithrix |
| 15 | Hafnia | unclassified Lachnospiraceae | EtOH8 |
| 16 | Dialister succinatiphilus | Blautia | rc4-4 |
| 17 | Lachnospira | Agrobacterium | Beijerinckiaceae |
| 18 | Mycobacteriaceae | Clostridium cluster XVIII | Benedictia fragilis |
| 18 | Streptococcus sp. | Bacteroides nordii | Desulfobacteraceae |
| 20 | Erysipelotrichaceae | Ramularia | Dorea |
| 21 | Dorea | Xylaria | Paraprevotella xylaniphila |
| 22 | Bacteroides fragilis group | Leptotrichia trevisanii | Robinsoniella |
| 23 | Bacteroides stercoris | Actinomyces sp. oral taxon 170 | Enterobacteriaceae |
| 24 | Akkermansia | Coprococcus | Galactomyces |
| 25 | Kineothrix | Stenotrophomas maltophila | Citrobacter koseri |
| 26 | Klebsiella | Clostridium sp. | Epicoccum |
| 27 | Clostridium cluster XVIII | Anaerosporobacter | Candidatus phytoplasma |
| 28 | Megamonas | Lachnospiraceae | Eggerthella sinensis |
| 29 | Clostridium sp. | unclassified Lactobacillales | Clostridium cluster XIVb |
| 30 | Fusobacterium mortiferum | Fournierella | Prevotella pallens |
